# Characteristics of Envelope Genes in a Chinese Chronically HIV-1 Infected Patient With Broadly Neutralizing Activity

**DOI:** 10.3389/fmicb.2019.01096

**Published:** 2019-05-24

**Authors:** Dai Zhang, Sen Zou, Yuanyuan Hu, Jiali Hou, Xintao Hu, Li Ren, Liying Ma, Xiang He, Yiming Shao, Kunxue Hong

**Affiliations:** ^1^State Key Laboratory of Infectious Disease Prevention and Control, National Center for AIDS/STD Control and Prevention, Chinese Center for Disease Control and Prevention, Collaborative Innovation Center for Diagnosis and Treatment of Infectious Diseases, Beijing, China; ^2^The First Affiliated Hospital of Henan University of Traditional Chinese Medicine, Zhengzhou, China; ^3^Guangdong Provincial Institute of Public Health, Guangdong Provincial Center for Disease Control and Prevention, Guangzhou, China

**Keywords:** HIV-1, envelope gene, broadly neutralizing antibodies, glycosylation, diversity

## Abstract

Exploring the characteristics of the HIV-1 envelope glycoprotein (*env*) gene in a natural HIV-1 infected individual, with broadly neutralizing activity, may provide insight into the generation of such broadly neutralizing antibodies and initiate the design of an appropriate immunogen. Recently, a chronically HIV-1 infected patient with broadly neutralization activity was identified and a VRC01-class neutralizing antibody DRVIA7 (A7) was isolated from the patient. In the present study, 155 full length HIV-1 *env* gene fragments (including 68 functionally Env clones) were amplified longitudinally from the plasma of six time points spanning over 5 years in this donor. Viral features were analyzed by comparing Env clones of different time points, as well as 165 Chinese HIV-1 subtype B *env* sequences from HIV Sequence Database (Chinese B_database). Shorter V1 length, less potential glycan and a lower ratio of NXT: NXS in gp160 were observed in the first five time points compared to that from the last time points, as well that from the Chinese B_database. A sequence analysis and a neutralization assay of Env-pseudoviruses showed that the increasing diversity of *env* sequences in the patient was consistent with the appearance and maturation of A7 lineage antibodies. The potent neutralization activity and viruses that escaped from the neutralization of the concurrent autologous plasma, are consistent with higher residue variations at the antibody recognition sites. Almost all viruses from the plasmas were neutralization-resistant to VRC01 and A7 lineage antibodies. For a chronically HIV-1 infected individual over 10 years, we found that greater viral diversity, short V1 sequences and less potential N-linked glycosylation (PNGS) in V1, might be associated with the development of broadly neutralizing antibody responses.

## Introduction

HIV-1 was identified as the pathogen of acquired immunodeficiency syndrome (AIDS) three decades ago (Gottlieb and Schroff, 1981; Barre-Sinoussi et al., 1983; Gallo et al., 1983; Dalgleish et al., 1984); however, the development of efficient and safe vaccines is still under way (Moore, 2018; Burton, 2019). The extreme virus diversity contributes to the main challenge for HIV-1 vaccines development. HIV-1 contains four groups: M, O, N, and P, and the dominant group M is further subdivided into nine distinct subtypes and increasing circulating recombinant forms (Robertson et al., 2000; Richman et al., 2003; Hemelaar, 2012). Broadly neutralization antibodies (bNAbs) targeting Env can protect animal models from the challenge of SHIV-1, neutralize most global circulating strains, and accelerate elimination of HIV-1-infected cells (Lu et al., 2016; Julg et al., 2017), therefore, eliciting bNAbs is an important goal of HIV-1 vaccines. However, in numerous pre-clinical and clinical trials of HIV-1 vaccines, bNAbs have not been successfully induced thus far (Burton and Hangartner, 2016; Kwong and Mascola, 2018).

Despite immune strategies to prevent HIV-1 infection have not been discovered, a recent study showed that broadly neutralizing activity can be detected in about 50% of HIV-1 infected individuals (Hraber et al., 2014), indicating that the human immune system indeed has the ability to elicit a bNAbs response. Before 2009, only four bNAbs were available; b12, 2F5, 4E10, and 2G12 (Muster et al., 1993; Buchacher et al., 1994; Conley et al., 1994; Trkola et al., 1996). Recently, with the wide utilization of new technologies such as single cell antibody cloning techniques, micro-neutralization assay, and B cell repertoire analysis (Simek et al., 2009; Walker et al., 2009, 2011; Huang et al., 2013; Zhu et al., 2013), hundreds of bNAbs were successively isolated from HIV-1-infected individuals (Burton and Hangartner, 2016; Wu and Kong, 2016). BNabs isolated in the natural HIV-1 infection provide a prototype that could be elicited by vaccines (Van Gils and Sanders, 2013; Bonsignori et al., 2017; Haynes and Mascola, 2017). A few studies tracing the evolution of bNAbs from the time of HIV-1 infection have revealed that viral and antibody evolution led to the induction and maturation of the bNAbs lineage (Liao et al., 2013; Gao et al., 2014; Bonsignori et al., 2016). However, given the huge diversity of HIV-1 and high complexity of the interaction between HIV-1 and the immune system, the development pathway of bNAbs was not all identical, even for a class of bNAbs in different individuals (Zhou et al., 2015). Thus, further exploring the general characteristics underlying the development of bNAbs would provide insights into efficient vaccines.

In our previous study, a long-term non-progressor (LTNP) DRVI01 with broadly neutralization activity was identified (Hu et al., 2012), and DRVIA7(A7), a VRC01-like broadly monoclonal neutralizing antibody targeting CD4 binding site (CD4bs) was isolated from this patient (Kong et al., 2016). Systematic analysis of the development of A7 over 5 years showed that the heavy chain of the antibody rapidly matured within 2 years, while the barrier of glycans of the gp120 protein blocked the development of the light chain of the antibody. However, the viral Env characteristics of DRVI01 have not been elaborated in detail.

In the present study, 155 full length HIV-1 *env* gene fragments were amplified longitudinally from the DRVI01 plasma of six time points spanning 5 years. Viral features were analyzed by comparing Env clones of different time points, as well as 165 Chinese HIV-1 subtype B Env sequences from the HIV Sequence Database (Chinese B_database). Sixty-eight functional Env clones were expressed as pseudoviruses to test neutralization sensitivities to autologous plasmas, representative bNAbs and A7 lineage reconstituted antibodies, respectively. The mutations of critical residues in the contact region of VRC01 were also analyzed. The results showed that for a chronically HIV-1 infected individual over 10 years, the greater viral diversity, short V1 sequences and less potential N-linked glycosylation (PNGS) in V1, might be associated with the development of broadly neutralizing antibody responses.

## Materials and Methods

### Study Subject

The samples described in this study were collected from an HIV-1-infected Chinese patient, DRVI01 who became HIV-1 infected by clade-B’ strain during commercial plasma donation between 1992 and 1995 (Hu et al., 2012; Kong et al., 2016). We collected the blood sample of the patient every 6 months between 2005 and 2010. The patient was antiretroviral treatment (ART)-naive, and the range of viral load of six time points ranged from 74,200 to 310,000 copies/ml and the CD4+ T cell count ranged from 335 to 769 cells/μl (Table 1). DRVI01 was identified as a broadly cross-reactive neutralizer, whose plasma exceeded a 95% neutralizing breadth against a panel of 25 viruses at all six time points. From PBMC of the subject, five neutralizing antibodies (NAbs) with limited neutralization breadth (all <40% breadth), including a VRC01-like neutralizing antibody DRVIA7, were isolated (Kong et al., 2016). The study was reviewed and approved by the Institutional Review Board of the National Center for AIDS/STD Control and Prevention, Chinese Center for Disease Control and Prevention. The subject provided written informed consent before blood and data collection.

**Table 1:** Characteristics of DRVI01 donor

Sample date	CD4+ T cells (cell/μl)	Viral load (copies/ml)	Mean *env* distance ± SD (%)	No. of *env* sequences	No. of functional Env clones
2005-7-12	475	1.27E+05	3.06 ± 1.35	29	14
2005-10-18	438	2.28E+04	3.17 ± 1.21	25	12
2006-4-6	335	7.42E+04	3.48 ± 1.63	34	15
2008-3-20	747	3.29E+05	4.79 ± 2.04^a^	32	18
2009-5-12	512	2.68E+05	5.26 ± 2.22^b^	14	N/A
2009-12-25	769	3.10E+05	5.36 ± 2.36^c^	21	9

*^a,b,c^Compared with 2006-4-6, 2005-10-18, and 2005-7-12, all P< 0.05.*

### Viral RNA Extraction, cDNA Synthesis, and Single-Genome Amplification

Viral RNA was extracted from the plasma using a QIAamp viral RNA mini kit (Qiagen, Valencia, CA) and subjected to first-strand cDNA synthesis immediately using the SuperScript III reverse transcriptase (Invitrogen Life Technologies, Grand Island, NY). Single-genome amplification (SGA) of the full-length gp160 gene was performed as described previously (Wu et al., 2012). Briefly, the synthesized cDNA was serially diluted and distributed in replicates of 12–16 PCRs in Thermo Grid 96-well plates, to identify a dilution where PCR-positive wells constituted about 30% of the total number of reactions. The SGA criteria of fewer than 30% positive results was acquired, and most of the wells contained amplicons derived from a single cDNA molecule in the suitable dilution.

### DNA Sequencing, Alignment, and Phylogenetic Analyses

SGA products were sequenced on an ABI 3770 Sequencer (Applied Biosciences). The full-length gp160 gene fragments for each amplicon were assembled and edited using Sequencher 4.1 (Gene Codes, Ann Arbor, MI). All chromatograms were inspected for sites of mixed bases (double peaks), which would provide evidence of priming from more than one template or the introduction of a PCR error in early cycles. Any sequence with evidence of double peaks was excluded from further analysis.

Phylogenetic and evolutionary analysis was conducted using MEGA 6. The *env* sequences were aligned using Gene Cutter.^1^ The nucleotide sequences together with B.CN.RL42.U71182, a Chinese B’ reference, were initially aligned and then checked by hand using BioEdit. The protein phylogenetic tree was built by the Neighbor-Joining method with the Jones–Taylor–Thornton model. After gap striping, the nucleotide phylogenetic tree was reconstructed by the maximum-likelihood method with GTR+Γ4+I substitution model. The reliability of internal nodes was assessed by a bootstrap test (1000 replicates). Genetic diversity of the Env variants from all time points was indicated as mean gene distances, which were calculated by MEGA 6.0 with the Bootstrap method and Kimura 2-parameter model.

### Variable Region Length and Gp160 PNGS Analyses

After the amino acid sequence alignment, the variable region length and the number of PNGS were determined using the online tool Variable Region Characteristics for V1, V2, V3, V4, V5.^2^ For comparison, a set of pre-aligned 165 HIV-1 subtype B Env protein sequences from China (Chinese B_database) were downloaded from the Los Alamos HIV database^3^ on June 15, 2017. The criteria for this data set were subtype B, intact gp160 sequences, Chinese, and one sequence per donor. The comparison of length variation and the V1 (aa 131–149), V2 (aa 158–197), V3 (aa 296–331), V4 (aa 385–418), and V5 (aa 460–471) loops in gp120 between different time points and the Chinese B_database were calculated by counting the number of amino acids. The number of PNGS and number of NXT or NXS motifs (X is any amino acid residue except proline) were identified using the N-Glycosite at the Los Alamos HIV database website.^4^

### Antibodies Used in the Study

BmAbs PGT121, PGT135, 2G12, 10E8, 12A21, and VRC01 were received from NIH AIDS Research and Reference Reagent Program (Trkola et al., 1996; Zhou et al., 2010; Walker et al., 2011; Huang et al., 2012, 2016). The heavy chains of A7 reconstituted antibodies were derived from DRVIA7H variants of 2006 and 2009, the light chains of antibodies were from a VRC01 light chain and 2009 DRVIA7L repertoire (Kong et al., 2016). We selected four reconstituted A7 antibodies in addition to DRVIA7 with a neutralization breadth ranging from 24 to 88% and the characteristics of A7 antibodies referred to Kong et al. (2016).

### Pseudovirus Preparation, Titration, and Neutralization Assays

Pseudoviruses were prepared, titrated as previously described (Li et al., 2005). Briefly, exponentially dividing 293T cells were cotransfected with Env/Rev expression plasmid and an Env-deficient HIV-1 backbone vector (pSG3△Env). Pseudovirus-containing supernatant was harvested 48 h post-transfection, and filtered (0.45 μm pore size) and single-use aliquots (1 ml) were stored at -80°C. The 50% tissue culture infectious dose (TCID50) of a single-thawed aliquot of each pseudovirus batch was determined in TZM-b1 cells.

Neutralization was measured as a reduction in Luc reporter gene expression after a single round of virus infection in TZM-bl cells, as described previously (Li et al., 2005). Briefly, 200 TCID50 of pseudovirus was incubated with serial threefold dilutions of plasma sample or antibodies for 1 h at 37°C. Freshly TZM-b1 cells were added. One set of control wells received cells only, and another set received cell plus pseudovirus. Following 48 h incubation, 150 μl culture was removed and 100 μl luciferase reporter gene assay system reagent (Bright-Glo; Promega) was added and incubated 2 min. 150 μl lysate from each well was transferred to 96-well black solid plates for measurement of luminescence using a luminometer (PerkinElmer Life Sciences). The 50% inhibitory dose (ID50) was defined as either the plasma dilution or sample concentration at which relative luminescence units (RLU) were reduced 50% compared to virus control wells.

### Statistical Analyses

SPSS software was used in the data analyses. Differences in the number of amino acids and potential N-linked glycans in the Env protein were compared using a one-way ANOVA, and the independent-samples *T* test was used between groups. Differences were considered significant if *P*< 0.05. The two-sided Fisher’s exact test was used to determine the difference of relative loss of specific PNGS between groups.

## Results

### Phylogenetic Analyses of the GP160 Genes

Env protein is the major target of NAbs. HIV-1 can escape the neutralization of NAbs with substitution, insertion or deletion in Env. To examine the Env evolution in DRVI01, SGA was used to isolate *env* genes from plasma samples. Around 20 (14–34) intact *env* genes (about 10 functional) were derived from each time point (Table 1). The virus diversity increased gradually from 2005-7 to 2009-12, and compared with those of the first three time points, significantly higher *env* diversification were found in the later three time points (Table 1).

**FIGURE 1:**
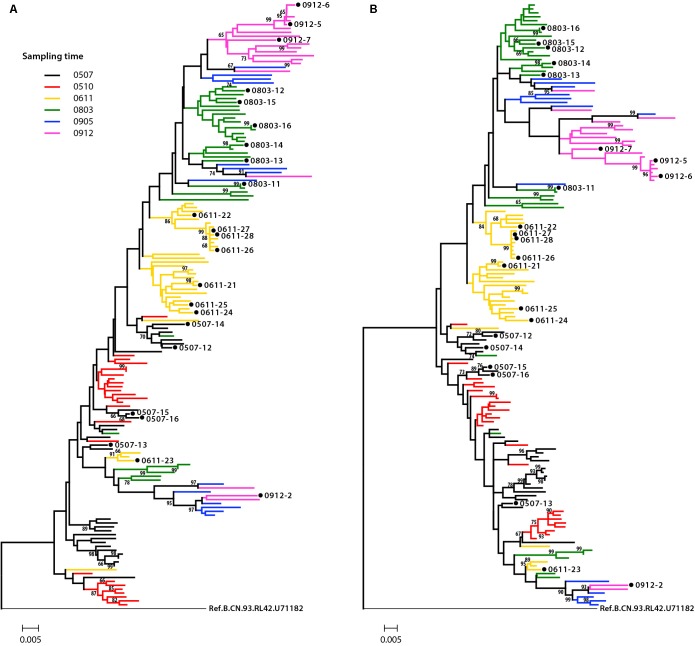
Phylogenetic tree of HIV-1 envelope sequences. A total of 155 gp160 sequences from an HIV-1-infected donor DRVI01 were aligned with the reference sequence B.CN.RL42.U71182. The tree was constructed based on sequence distance and rooted at B.CN.RL42.U71182 for visualization. **(A)** The protein phylogenetic tree was built by the Neighbor-Joining method with the Jones–Taylor–Thornton model. **(B)** The nucleotide phylogenetic tree was reconstructed by the maximum-likelihood method with GTR+Γ4+I substitution model. The tree showed that a main viral population and a minor viral population have evolved in parallel in the patient for more than 4.5 years, and the latter is not eliminated over time. The gp160 sequences were color coded as follows: black, June 2005; red, October 2005; yellow, 2006; green, 2008; blue, May 2009; purple, December 2009. The horizontal branch scale is indicated for each tree.

Phylogenetic analysis was performed to examine the relationship of these Env sequences, both protein sequence tree (Figure 1A) and nucleotide sequence tree (Figure 1B) displayed similar clustering mode. Most of the sequences formed distinct time-specific lineages, with a fraction of sequences intermingled. The phylogenetic trees, especially the nucleotide sequence one, showed that a main viral population and a minor viral population evolved in parallel in the patient for more than 4.5 years, and the latter was not eliminated over time. The gene distance of all time points for all sequences is 0.028876 ± 0.002026 (mean ± SE). The distances of minor branch and main branch were 0.01963 ± 0.00144, 0.02372 ± 0.00152, respectively. Compared with the minor population, the main viral population showed higher diversity, which may reflect the better adaptation of HIV-1 in response to host selective pressures (Figure 1B). However, no differences in neutralization sensitivity of viruses between the main and minor branches were observed (Table 3).

### Significantly More Potential PNGS and the NXT:NXS Ratio of Gp160 at the Later Time Points

**FIGURE 2:**
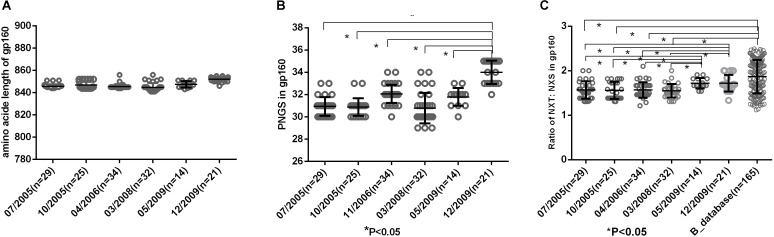
**(A)** Comparison of amino acid length of gp160, which indicted no significant differences in amino acid length of gp160 among different time points. **(B)** Comparisons of glycan number of gp160 in six time points, which showed an increasing glycan number from 07/2005 to 12/2009 and the glycan number in gp160 from 12/2009 was significantly higher than the first five time points (07/2005–05/2009) (all *P* < 0.05). **(C)** Ratio of NXT:NXS of gp160 in six time points and Chinese B_database. NXT: NXS ratios in gp160 from the first four time points were significantly lower than those from two time points in 2009 and Chinese B_database (all *P* < 0.05).

Env uses multiple mechanisms to escape from host immune response, including amino acid substitutions, insertions in the variable domains and increasing PNGS on its outer surface. In this study, we compared the amino acid length, PNGS and ratio of NXT:NXS at the six time points to observe the features of gp160. The results showed the length of gp160 was relatively constant (Figure 2A), however, the number of PNGS in gp160 were significantly more at 2009-12 time point than those at the first five time points (Figure 2B). It has been reported that NXS motifs have a two to three times lower probability of becoming glycosylated than NXT motifs (Kaplan et al., 1987; Gavel and Heijne, 1990). The actual extent of glycosylation of a given Env molecule may therefore not entirely equal to the total number of PNGS. In the study, we found the ratio of NXT: NXS in gp160 at the first four time points (07/2005–03/2008) were significantly lower than those at the last two time points (05/2009, 12/2009) and the Chinese B_database (all *P*< 0.05) (Figure 2C). Altogether, amino acid substitutions and increasing PNGS are likely the main ways that viruses adapt the host immune response in the patient.

### Shorter V1 Length and Fewer PNGS in V1 at the First Five Time Points

**FIGURE 3:**
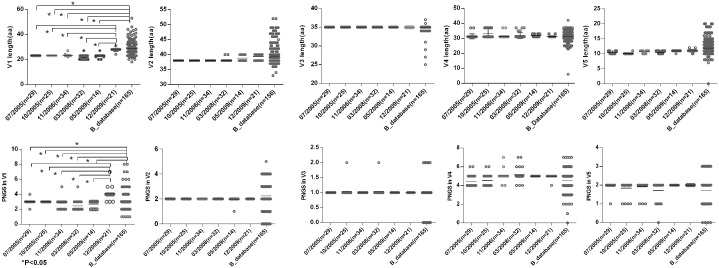
Comparisons of sequence length and glycan number between DRVI01 and Chinese B-database. Compared with that of Chinese B_database, the V1 region lengths of time points 07/2005, 10/2005, 04/2006, 03/2008, 05/2009 were significantly shorter (all *P* < 0.05); the V1 length of 12/2009 was the longest among six time points. As well, the glycan numbers in V1 of Chinese B_database and12/2009 were more than that of five time points (all *P* < 0.05). The V2 length of two time points in 2009 and Chinese B_database were slightly increased than that of the first four time points.

To observe the features of the Env sequences from different time points, we further compared variable regions length and numbers of PNGS and the ratio of NXT:NXS from six time points as well as the Chinese B_database. The results showed that a shorter V1 region and fewer numbers of PNGS in V1 at the first five time points compared with those at 2009-12 time point and Chinese B_database (Figure 3). Heavily glycosylated V1V2 loop locates in the apex of Env spike were observed, and the loop can obstruct the exposure of co-receptor and CD4 binding sites (Wyatt et al., 1995; Rusert et al., 2011). In general, an increase of the length of V1V2 and the number of PNGS would help viruses escape autologous antibody neutralization and shield the more conserved domains associated with receptor binding (Van Gils et al., 2011; Wu et al., 2012). Our results were inconsistent with previous reports, as the length of theV1V2 loop was not significantly increased in the subject, after a HIV-1 infection over 10 years. Some studies considered that early HIV-1 variants with shorter variable V1V2 loop correlated with the development of later cross-reactive neutralizing activity (Rademeyer et al., 2007; van den Kerkhof et al., 2013). At the first four time points, the lower ratio of NXT:NXS indicated lower probability of glycosylation, combining with shorter V1 length and fewer PNGS may favor exposure of interior conservative epitopes on Env and the binding of the B cell receptor to interior epitopes.

### Increasing Env Diversity Consistent With the Development of A7 Antibodies

**FIGURE 4:**
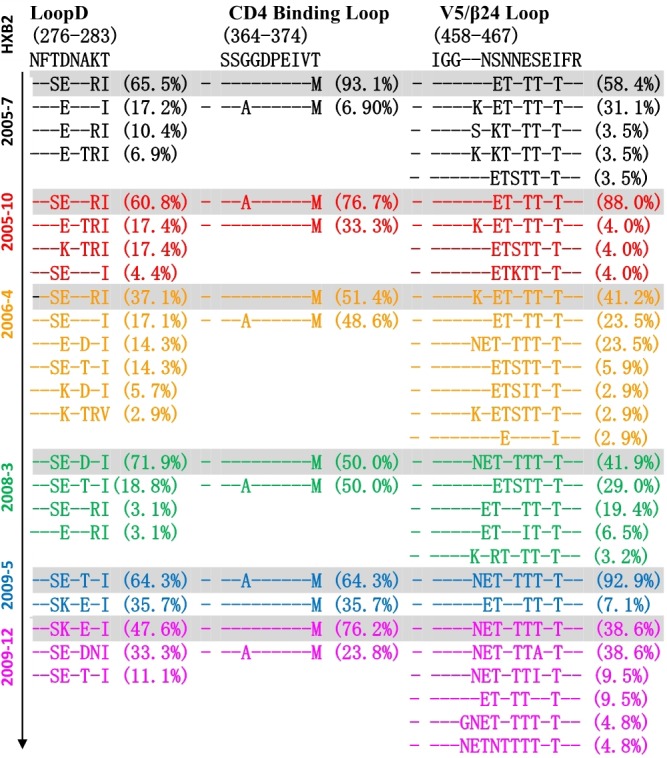
Amino acid evolutionary modes located in the contact regions of gp120 and VRC01 including loop D (276–283), CD4 binding loop (364–374) and V5/β24 loop (458–467) in six time points (HXB2 numbering is indicated). Different amino acids sequences in three contact regions were sorted and denoted percentage in 155 Env sequences of six time points, and – (dashed) represents same residues with HXB2. The sequences were color coded as follows: black, June 2005; red, October 2005; yellow, 2006; green, 2008; blue, May 2009; purple, December 2009. The dominant variants presented more mutations in loop D at later time point, and the highest diversification of loop D was found at 2006-04 time point. CD4 binding loop exhibited lower diversification spanning all six time points, and more diversification in V5 was found in the dominant variants at later time point.

Co-evolution of the virus-antibody in the HIV infection lead to the induction and development of bNAbs, and Env diversification in contact residues of bNAbs preceded the development of the neutralization breadth (Liao et al., 2013; Bonsignori et al., 2016). Therefore, the characteristics of evolutionary modes of contact residues of gp120 and VRC01 in HIV infection could provide valuable insight in designing the sequential immunogens. Loop D plays an important role in the interaction of VRC01 and gp120, and recurrent mutations in loop D were found in VRC01-resistant viruses (Zhou et al., 2010; Li et al., 2011). By longitudinally tracking the evolution of Env from six time points, we found that the dominant variants presented more mutations in loop D at a later time point (Figure 4). Interestingly, the highest diversification of loop D was found at the 2006-04 time point, and the time coincided with the emergence of the A7 lineage, suggesting that the diversification of loop D could be associated with the induction of the A7 lineage. The CD4 binding loop was one of the critical contact regions of VRC01 and gp120. Mutations in this region could result in the loss of the capability of virus infection. In our study, the CD4 binding loop exhibited lower diversification, spanning all six time points. The V5/β24 loop was another contact region of VRC01 and gp120. Variants in the region were observed in the VRC01 resistant isolates (Zhou et al., 2010; Li et al., 2011). In line with our observation in loop D, most of the mutations in V5/β24 were focused in the tip of the V5 loop, and more diversification in V5 were found in the dominant variants at a later time point (Figure 4).

Altogether, three main contact regions of VRC01 and gp120 presented multiple mutations at all six time points. Higher residue variation at antibody recognition sites indicated that the viral and antibody evolution might lead to induction and maturation of A7 lineage antibodies.

### Antibody-Based Selection Pressure Driving Ongoing Viral Evolution

Plasma from six time points of DRVI01 exhibited potent neutralization activity and can neutralize over 95% of viruses in a panel of 25 pseudoviruses (Kong et al., 2016). To observe the interaction between viruses and autologous plasmas, the neutralization sensitivity of the functional Env variants of five time points was measured against autologous plasma samples (Table 2). In line with a previous observation, autologous plasmas can potently neutralize viruses from earlier time points but cannot neutralize viruses from concurrent and later time points (Bunnik et al., 2008; Moore et al., 2009a,b; Wu et al., 2012). The results indicated that even a robust immune response can efficiently neutralize autologous viruses; HIV-1 managed to escape the neutralization of NAbs by mutations over time. Consistent with phylogenetic analysis, antibody-based selection pressure drove the ongoing viral evolution.

**Table 2:** Neutralization sensitivity of the Env pseudoviruses against autologous plasmas

Pseudoviruses	Autologous neutralization ID50				
	2005-7-12	2005-10-18	2006-4-6	2008-3-20	2009-12-25
*2005-7-12*					
0507-12	<20	172	187	413	708
0507-13	<20	138	391	206	1086
0507-14	<20	158	262	310	852
0507-15	<20	98	664	553	1407
0507-16	<20	155	318	698	759
Potency (GMTs)	<20	135	384	396	997
Breadth (*n* = 5)	0 (0)	5 (100%)	5 (100%)	5 (100%)	5 (100%)
*2006-4-6*					
0604-21	<20	<20	<20	232	603
0604-22	<20	<20	<20	258	1541
0604-23	<20	<20	<20	328	1165
0604-24	<20	<20	<20	89	1254
0604-25	<20	<20	<20	99	743
0604-26	<20	<20	<20	201	865
0604-27	<20	<20	<20	176	711
0604-28	<20	<20	<20	58	943
Potency (GMTs)	<20	<20	<20	139	810
Breadth(*n* = 8)	0 (0)	0 (0)	0 (0)	8 (100%)	8 (100%)
*2008-3-20*					
0803-11	<20	<20	<20	<20	654
0803-12	<20	<20	<20	<20	764
0803-13	<20	<20	<20	<20	773
0803-14	<20	<20	<20	<20	854
0803-15	<20	<20	<20	<20	494
0803-16	<20	<20	<20	<20	1125
Potency (GMTs)	<20	<20	<20	<20	741
Breadth(*n* = 6)	0 (0)	0 (0)	0 (0)	0 (0)	6 (100%)
*2009-12-25*					
0912-1	<20	<20	<20	<20	<20
0912-2	<20	<20	<20	<20	<20
0912-3	<20	<20	<20	<20	<20
0912-4	<20	<20	<20	<20	<20
0912-5	<20	<20	<20	<20	<20
0912-6	<20	<20	<20	<20	<20
0912-7	<20	<20	<20	<20	<20
Potency (GMTs)	<20	<20	<20	<20	<20
Breadth(*n* = 7)	0 (0)	0 (0)	0 (0)	0 (0)	0 (0)

*^a^The neutralizing potency is measured as ID50 in dilution of the plasmas samples. The plasmas dilution values >1000 are highlighted in red, 200∼1000 in yellow, and 20∼200 in green. ^b^GMT: geometric mean titer. ^c^The neutralizing breadth is calculated as the percentage of viruses neutralized with ID50 > 20. ^d^Pseudoviruses were named by the time point + number.*

### Neutralization Sensitivity of the Pseudoviruses Against the bNAbs Consistent With Variation in Critical Residues

The neutralization sensitivity of the Env pseudoviruses from different time points was tested against six bNAbs including PGT121, PGT135, 2G12, VRC01, 10E8, and12A21; and the mutations of critical residues in these bNAbs epitopes were analyzed for each Env variant (Table 3 and Supplementary Table 1). Almost all pseudoviruses were found to be resistant to VRC01 and 12A21, suggesting the presence of CD4bs antibodies pressure over the study period, which was consistent with the strong CD4bs antibodies specificity and isolation of a VRC01-class antibody A7 from the subject (Kong et al., 2016). Binding interface of VRC01 on gp120 largely located in Loop D, CD4 binding loop and V5/β24; viruses can abrogate VRC01-mediated neutralization by key residue mutations in these regions (Zhou et al., 2010; Li et al., 2011; Lynch et al., 2015). In our study, all the Env clones from DRVI01 presented mutations in the contact regions for VRC01, in particular, residues in loop D and the V5/β24 loop displayed continuous mutations (Supplementary Table 1).

**Table 3:** Neutralization sensitivity of the Env pseudoviruses against bNAbs and reconstituted A7 lineage antibodies

Pseudoviruses	IC_50_ (μg/ml)										
	PGT121	PGT135	2G12	VRC01	10E8	12A21	gDRVI01-H68+ VRC01 L	gDRVI01-H69+ VRC01 L	DRVIA7H+ gDRVIA7-L40	DRVIA7H+ gDRVIA7-L42	DRVIA7
0507-12	0.54	1.56	0.18	>50	0.72	>50	>50	>50	>50	>50	>50
0507-13	0.42	0.34	0.30	>50	0.65	>50	>50	>50	>50	>50	>50
0507-14	0.02	2.02	0.17	>50	0.71	>50	>50	>50	>50	>50	>50
0507-15	0.61	0.97	0.20	>50	0.13	>50	>50	>50	>50	>50	>50
0507-16	0.44	0.21	0.12	0.84	0.09	1.43	>50	>50	>50	>50	>50
0604-21	0.56	1.81	0.20	>50	0.15	>50	>50	>50	>50	>50	>50
0604-22	0.51	2.42	0.25	>50	0.65	>50	>50	>50	>50	>50	>50
0604-23	0.27	0.12	0.18	>50	2.29	>50	>50	>50	>50	>50	>50
0604-24	0.70	1.19	0.14	>50	3.67	>50	>50	>50	>50	>50	>50
0604-25	0.26	0.33	0.14	>50	0.32	>50	>50	>50	>50	>50	>50
0604-26	0.17	0.16	0.13	>50	0.18	>50	>50	>50	>50	>50	>50
0604-27	4.12	0.12	0.12	>50	0.81	>50	>50	>50	>50	>50	>50
0604-28	0.02	0.30	0.41	>50	0.12	>50	>50	>50	>50	>50	>50
0803-11	0.12	0.38	0.55	>50	1.10	>50	>50	>50	>50	>50	>50
0803-12	0.58	0.24	0.97	>50	0.11	>50	>50	>50	>50	>50	>50
0803-13	0.13	0.17	0.34	>50	0.59	>50	>50	>50	>50	>50	>50
0803-14	0.17	0.20	0.32	>50	0.34	>50	>50	>50	>50	>50	>50
0803-15	0.63	0.35	0.48	>50	0.61	>50	>50	>50	>50	>50	>50
0803-16	0.92	0.37	0.18	>50	0.09	>50	>50	>50	>50	>50	>50
0912-1	2.44	2.94	0.65	>50	0.30	>50	>50	>50	>50	>50	>50
0912-2	1.65	0.37	0.92	>50	0.07	>50	>50	>50	>50	>50	>50
0912-3	4.04	0.22	2.44	>50	0.19	>50	>50	>50	>50	>50	>50
0912-4	1.37	3.77	0.28	>50	0.03	>50	>50	>50	>50	>50	>50
0912-5	0.18	0.21	0.17	>50	0.19	>50	>50	>50	>50	>50	>50
0912-6	0.58	1.37	0.40	>50	0.31	>50	>50	>50	>50	>50	>50
0912-7	1.49	1.90	0.41	>50	0.18	>50	>50	>50	>50	>50	>50

*^a^The neutralizing potency is measured as IC_50_ in μg/ml of the monoclonal antibodies. Values < 0.2μg/ml are highlighted in red, 0.2∼2μg/ml in yellow, and 2∼20μg/ml in green. ^b^The characteristics and neutralization data of A7 lineage reconstituted Abs refer to the study from Kong et al. (2016). ^c^Pseudoviruses were named by the time point + number.*

It has been reported that single or combined mutations at position 279/281/282 were the common escape pathway of HIV-1 under immune pressure of VRC01-class antibodies (Li et al., 2011; Lynch et al., 2015). In our study, the 279E mutation was mainly observed at the first three time points, the position gradually shifted to E279K at later time points. Residues A281 was relatively constant at the first two time points, a mutation in position 281 emerged at the third time point, and the position was substituted to D/E/T in all of the isolates at three later time points. In contrast, a K282R mutation presented at the first two time points, the position K282 was relatively constant at the later four time points. Mutations and insertion within the V5/β24 loop were mainly observed in the tip of V5 loop, which had been considered to be unchangeable to neutralization sensitivity of VRC01. For Env clones from DRVI01, more substitutions and insertions in V5 loop were found at the later four time points with a trend of gradually increasing diversification over time. Almost all of Env clones presented a mutation N462T, and combined with N460 produced a PNGS, which may be a potential obstruction to the interaction between VRC01 and Env (Guo et al., 2012). Altogether, continuous mutations within loop D and the V5 loop resulted in viruses evasion from VRC01-class antibodies, more changes in loop D and the V5 loop were attended by the emergence of the A7 antibody, suggesting that the diversification in contact residues may be associated with the induction and development of A7 lineage antibodies.

In contrast, we found that almost all pseudoviruses were highly sensitive to PGT121, PGT135, 2G12, and 10E8 (most of IC_50_ < 0.5 μg/ml), which were consistent with the observed indistinguishable variants in critical residues of these bNAbs at different time points (Table 3 and Supplementary Table 1).

### All Env Pseudoviruses Were Resistant to DRVIA7 Lineage Antibodies

DRVIA7(A7), a VRC01-like antibody, was isolated from the patient’s PBMC of 2009 time point. Longitudinal tracing of the B cell repertoires across 2006, 2008, and 2009 showed that the heavy chain of the antibody matured rapidly in 2 years, and the functional precursors of the A7 lineage heavy chain might emerge in 2006 (Kong et al., 2016). To search for the functional Env with the binding ability to the early stage A7 lineage, we tested neutralization sensitivity of Env pseudoviruses to five A7 lineage reconstituted antibodies. Our results showed that all of the Env pseudoviruses from DRVI01 were resistant to five A7 reconstituted antibodies (Table 3), which indicated that functional Env clones binding to the A7 lineage may be relatively rare. The results were consistent with the sequence analysis that all of the isolates from DRVI01 contained mutations in critical contact regions of VRC01 (Supplementary Table 1).

## Discussion

In a previous study, a VRC01-like antibody A7 was isolated from an HIV-1-infected individual with potent neutralization activity (Kong et al., 2016). Longitudinal analysis of B cell repertoires across 2006, 2008, and 2009 revealed that the A7 heavy chain rapidly matured within 2 years, reached peak in 2008, and declined in 2009 due to stalled light chain maturation. In this study, we isolated 155 full length Env sequences (including 68 functional Envs) from six sequential plasma samples of the patient, using SGA, to explore the characteristics of Env associated with A7 development.

Diversification of Env was associated with the induction and maturation of bNAbs, which provided a wealth of antigenic stimulation for the immune system, and increased the probability of activating B cell precursors of bNAbs (Wibmer et al., 2013; Doria-Rose et al., 2014; Bhiman et al., 2015). In the present study, although neutralization breadth had already reached a plateau in 2005 (Kong et al., 2016), the continuous evolution of NAbs was observed to be attended by an active mutation of the virus through six time points. Gene distance and phylogenetic analysis of gp160 sequences exhibited a trend of continuous evolution. Diversification of Env gradually increased from time point 2005-10, and diversification of Env in time points 2008-3, 2009-5, and 2009-12 was significantly higher than that of the first three time points, which was consistent with emergence of A7 lineage precursors in 2006. The results indicated a continuous active interaction between the virus and the immune system in the patient.

Sequence analysis showed a shorter V1 region, lower PNGS and a lower ratio of NXT:NXS in the first five time points compared with those in time point 2009-12 and the Chinese B_database. V1V2 loop locates in the apex of the functional Env spike, and displays high amino acid variability (Wyatt et al., 1995). HIV-1 may escape from neutralization by means of a conformational mask, glycan shield, and so on (Van Gils et al., 2011). Deletion of V1V2 loop or diminishing the glycan in V1V2 could increase the neutralization sensitivity of autologous plasma and NAbs, indicating that the V1V2 loop plays an important role in the shield of the vulnerability site of the Env spike (Cao et al., 1997; Pinter et al., 2004; Bontjer et al., 2009). The Virus could escape the neutralization of NAbs by increasing the length of the V1V2 loop and number of the glycan in V1V2 (Van Gils et al., 2011; Wu et al., 2012). Some studies observed a correlation between shorter V1, lower PNGS and the induction of bNAbs, which could be explained by reducing the shield in inner vulnerability sites of the Env spike (Rademeyer et al., 2007; Bunnik et al., 2010; van den Kerkhof et al., 2013). Env V1 mutations were found to be adjacent to contact residues for CD4 and VRC01, insertions in V1 would inhibit the access to the CD4bs in the trimer (Liao et al., 2013). A shorter V1 region and lower PNGS retaining at the first five time points from DRVI01 may favor the development of Nabs.

The development of bNAbs was shown to correlate with continuous mutations directly in or adjacent to the NAbs/Env contact region, which allowed sufficient somatic hypermutation of BCR and focuses the B-cell response to the conserved vulnerability sites on Env (Sather et al., 2009; Klein et al., 2012; Liao et al., 2013). Loop D, CD4 binding loop and V5 loop play important roles in VRC01 binding with gp120 (Zhou et al., 2010; Li et al., 2011; Lynch et al., 2015). In the study, longitudinally tracking the evolution of DRVI01 *env* genes showed that more mutations presented in loop D and V5 regions over the five time points. All 155 Env clones contained three PNGS in positions 276, 460, and 463, which could obstruct the binding of the germlines of VRC01-class antibodies with the Env spike (Li et al., 2011; Guo et al., 2012; Wang et al., 2015). Previous longitudinal tracing of the A7 lineage development inferred the birth date of the A7 precursor B cells shortly before time point 2006 (Kong et al., 2016), it could be postulated that the Env variants prior to that time point may activate the precursor B cells of A7 lineage, though it could not be isolated by SGA in our study. Additionally, we observed that the diversification and mutations in loop D and the V5 region began to arise from the 2006 time point, variants with different modes alternatively appeared over time. The results indicated that viruses escaping neutralization of VRC01-class antibodies, by means of mutating NAbs contact residues, could drive the breadth of NAbs.

The robust immune responses driving continuous escape mutants facilitated the development of bNAbs (Liao et al., 2013; Wibmer et al., 2013; Gao et al., 2014; Bonsignori et al., 2016). In the present study, neutralization sensitivity of the Env isolates against autologous plasma and a few well-known bNabs was analyzed to observe virus-antibody interactions. The plasma of DVRI01 presented potent and broad neutralization activity (Hu et al., 2012; Kong et al., 2016). As expected, almost all Env isolates from the patient escaped neutralization by concurrent autologous plasma. The results indicated that the strong autologous neutralizing selection pressure continuously drove the viruses to escape. Neutralization sensitivity of the Env pseudoviruses against a few bNAbs showed that all Env clones were VRC01-resistant, suggesting the presence of strong immune pressure from the VRC01-class antibodies in the patient. In contrast, PGT121, PGT135, 10E8, and 2G12 which target the glycans in the V3 region, MPER, and the glycans in outer gp120, respectively, could potently neutralize all Env clones, which were in line with the conserved critical residues of these bNAbs epitopes over the study period, indicating a lack of immune pressure of the above four bNAbs in the patient.

The functional Env clones binding germline precursors of bNAbs have been considered as potential virus strains initiating the development of bNAbs. In the present study, however, all Env pseudoviruses derived from DRVI01 plasma were proven to be neutralization-resistant toward the five reconstituted A7 lineage antibodies, suggesting that the virus strains associated with the development of the A7 lineage were not dominant. Our previous analysis of the germline gene usage also displayed that IgHV1-2, the germline family of DRVIA7 heavy chain, were significantly lower than IgHV4-34 and IgHV4-39 across the 2006, 2008, and 2009 time points, indicating that DRVIA7 did not constitute a major lineage within the repertoire (Kong et al., 2016). Dynamic antibody evolution revealed that A7 lineage precursors emerged in 2006, but all viruses isolated before 2006 were resistant to A7 lineage antibodies, suggesting that immune pressure of VRC01-class antibodies already presented prior to 2006. The results were consistent with an Env sequences analysis which showed many mutations already presented in loop D and V5 at the two 2005 time points. Therefore, the immune pressure of VRC01-class antibodies presented prior to the timeframe studied could result in failure in isolating the Env clones capable of binding A7 lineages.

In summary, we acquired 155 intact *env* sequences from a Chinese chronically HIV-1-infected individual with potent neutralization activity. Both the sequence and neutralization analysis showed that the gradually increasing diversification of the Env sequences was associated with the development of the A7 lineage; the robust neutralization activity of plasmas and the escaped mutants from autologous plasmas were consistent with more mutations in the contact region of Nabs, which suggests a continuous co-evolution of Env and Nabs. Additionally, sequences analysis observed a few characteristics that could facilitate the recognition of CD4bs antibodies, which contained shorter V1, lower PNGS and a ratio of NXT:NXS at the first five time points.

There were several limitations in the study. First, the subject DRVI01 was infected over 10 years, the early samples of infection were unavailable for the study. Second, the precursor of the heavy chain of the A7 lineage was inferred to emerge before 2006, but the unmutated common ancestor (UCA) had not been identified in previous studies. Third, we could not identify the Env variants capable of binding the A7 lineage antibodies.

## Ethics Statement

The study was reviewed and approved by the Institutional Review Board of the National Center for AIDS/STD Control and Prevention, Chinese Center for Disease Control and Prevention. The subject provided written informed consent before blood and data collection.

## Author Contributions

DZ, KH, and YS designed the study, analyzed the data, and edited the manuscript. DZ, SZ, YH, and XH performed the *env* cloning, sequencing, and phylogenetic analysis. DZ, JH, SZ, XTH, and LR prepared the pseudoviruses and performed the neutralization assays. YS, LM, and KH developed the cohort and collected the samples.

## Conflict of Interest Statement

The authors declare that the research was conducted in the absence of any commercial or financial relationships that could be construed as a potential conflict of interest.

## Footnotes

1 https://www.hiv.lanl.gov/content/sequence/GENE_CUTTER/cutter.html

2 https://www.hiv.lanl.gov/content/sequence/VAR_REG_CHAR/index.html

3 https://www.hiv.lanl.gov/

4 http://www.hiv.lanl.gov/content/sequence/GLYCOSITE/glycosite.html

## References

[B1] Barre-Sinoussi F. Chermann J. Rey F. Nugeyre M. Chamaret S. Gruest J. (1983). Isolation of a t-lymphotropic retrovirus from a patient at risk for acquired immune deficiency syndrome (aids). *Science* 220 868–871. 10.1126/science.6189183 6189183

[B2] Bhiman J. N. Anthony C. Doriarose N. A. Karimanzira O. Schramm C. A. Khoza T. (2015). Viral variants that initiate and drive maturation of v1v2-directed HIV-1 broadly neutralizing antibodies. *Nat. Med.* 21 1332–1336. 10.1038/nm.3963 26457756 PMC4637988

[B3] Bonsignori M. Liao H. X. Gao F. Williams W. B. Alam S. M. Montefiori D. C. (2017). Antibody-virus co-evolution in HIV infection: paths for HIV vaccine development. *Immunol. Rev.* 275 145–160. 10.1111/imr.12509 28133802 PMC5302796

[B4] Bonsignori M. Zhou T. Sheng Z. Chen L. Gao F. Joyce M. G. (2016). Maturation pathway from germline to broad HIV-1 neutralizer of a CD4-mimic antibody. *Cell* 165 449–463. 10.1016/j.cell.2016.02.022 26949186 PMC4826291

[B5] Bontjer I. Land A. Eggink D. Verkade E. Tuin K. Baldwin C. (2009). Optimization of human immunodeficiency virus type 1 envelope glycoproteins with v1/v2 deleted, using virus evolution. *J. Virol.* 83 368–383. 10.1128/JVI.01404-08 18922866 PMC2612307

[B6] Buchacher A. Predl R. Strutzenberger K. Steinfellner W. Jungbauer A. (1994). Generation of human monoclonal antibodies against HIV-1 proteins; electrofusion and epstein-Barr virus transformation for peripheral blood lymphocyte immortalization. *AIDS Res. Hum. Retroviruses* 10 359–369. 10.1089/aid.1994.10.359 7520721

[B7] Bunnik E. M. Euler Z. Welkers M. R. Boesernunnink B. D. Grijsen M. L. Prins J. M. (2010). Adaptation of HIV-1 envelope gp120 to humoral immunity at a population level. *Nat. Med.* 16 995–997. 10.1038/nm.2203 20802498

[B8] Bunnik E. M. Pisas L. Van Nuenen A. C. Schuitemaker H. (2008). Autologous neutralizing humoral immunity and evolution of the viral envelope in the course of subtype b human immunodeficiency virus type 1 infection. *J. Virol.* 82 7932–7941. 10.1128/JVI.00757-08 18524815 PMC2519599

[B9] Burton D. R. (2019). Advancing an HIV vaccine; advancing vaccinology. *Nat. Rev. Immunol.* 19 77–78. 10.1038/s41577-018-0103-6 30560910 PMC6425752

[B10] Burton D. R. Hangartner L. (2016). Broadly neutralizing antibodies to HIV and their role in vaccine design. *Annu. Rev. Immunol.* 34 635–659. 10.1146/annurev-immunol-041015-055515 27168247 PMC6034635

[B11] Cao J. Sullivan N. Desjardin E. Parolin C. Robinson J. Wyatt R. (1997). Replication and neutralization of human immunodeficiency virus type 1 lacking the v1 and v2 variable loops of the gp120 envelope glycoprotein. *J. Virol.* 71 9808–9812. 9371651 10.1128/jvi.71.12.9808-9812.1997 PMC230295

[B12] Conley A. J. Kessler J. A. Boots L. J. Tung J. S. Arnold B. A. Keller P. M. (1994). Neutralization of divergent human immunodeficiency virus type 1 variants and primary isolates by IAM-41-2f5, an anti-gp41 human monoclonal antibody. *Proc. Natl. Acad. Sci. U.S.A.* 91 3348–3352. 10.1073/pnas.91.8.3348 7512731 PMC43574

[B13] Dalgleish A. G. Beverley P. C. L. Clapham P. R. Crawford D. H. Greaves M. F. Weiss R. A. (1984). The CD4 (t4) antigen is an essential component of the receptor for the aids retrovirus. *Nature* 312 763–767. 10.1038/312763a0 6096719

[B14] Doria-Rose N. A. Schramm C. A. Gorman J. Moore P. L. Bhiman J. N. DeKosky B. J. (2014). Developmental pathway for potent V1V2-directed HIV-neutralizing antibodies. *Nature* 509 55–62. 10.1038/nature13036 24590074 PMC4395007

[B15] Gallo R. C. Sarin P. S. Gelmann E. P. Robert-Guroff M. Richardson E. Kalyanaraman V. S. (1983). Isolation of human t-cell leukemia virus in acquired immune deficiency syndrome (aids). *Science* 220 865–867. 10.1126/science.6601823 6601823

[B16] Gao F. Bonsignori M. Liao H. X. Kumar A. Xia S. M. Lu X. (2014). Cooperation of B cell lineages in induction of HIV-1-broadly neutralizing antibodies. *Cell* 158 481–491. 10.1016/j.cell.2014.06.022 25065977 PMC4150607

[B17] Gavel Y. Heijne G. V. (1990). Sequence differences between glycosylated and non-glycosylated Asn-X-Thr/Ser acceptor sites: implications for protein engineering. *Protein Eng. Des. Sel.* 3 433–442. 10.1093/protein/3.5.433 2349213 PMC7529082

[B18] Gottlieb M. S. Schroff R. (1981). *Pneumocystis carinii* pneumonia and mucosal candidiasis in previously healthy homosexual men: evidence of a new acquired cellular immunodeficiency. *N. Engl. J. Med.* 305 1425–1431. 10.1056/NEJM198112103052401 6272109

[B19] Guo D. Shi X. Arledge K. C. Song D. Jiang L. Fu L. (2012). A single residue within the V5 region of HIV-1 envelope facilitates viral escape from the broadly neutralizing monoclonal antibody VRC01. *J. Biol. Chem.* 287 43170–43179. 10.1074/jbc.M112.399402 23100255 PMC3522310

[B20] Haynes B. F. Mascola J. R. (2017). The quest for an antibody-based HIV vaccine. *Immunol. Rev.* 275 5–10. 10.1111/imr.12517 28133795 PMC5384259

[B21] Hemelaar J. (2012). The origin and diversity of the HIV-1 pandemic. *Trends Mol. Med.* 18 182–192. 10.1016/j.molmed.2011.12.001 22240486

[B22] Hraber P. Seaman M. S. Bailer R. T. Mascola J. R. Montefiori D. C. Korber B. T. (2014). Prevalence of broadly neutralizing antibody responses during chronic HIV-1 infection. *AIDS* 28 163–169. 10.1097/QAD.0000000000000106 24361678 PMC4042313

[B23] Hu X. Hong K. Zhao C. Zheng Y. Ma L. Ruan Y. (2012). Profiles of neutralizing antibody response in chronically human immunodeficiency virus type 1 clade B′-infected former plasma donors from China naive to antiretroviral therapy. *J. Gen. Virol.* 93(Pt_10), 2267–2278. 10.1099/vir.0.043802-0 22791603

[B24] Huang J. Doria-Rose N. A. Longo N. S. Laub L. Lin C. L. Turk E. (2013). Isolation of human monoclonal antibodies from peripheral blood B cells. *Nat. Protoc.* 8 1907–1915. 10.1038/nprot.2013.117 24030440 PMC4844175

[B25] Huang J. Kang B. H. Ishida E. Zhou T. Griesman T. Sheng Z. (2016). Identification of a CD4-binding-site antibody to HIV that evolved near-pan neutralization breadth. *Immunity* 45 1108–1121. 10.1016/j.immuni.2016.10.027 27851912 PMC5770152

[B26] Huang J. Ofek G. Laub L. Louder M. K. Doria-Rose N. A. Longo N. S. (2012). Broad and potent neutralization of HIV-1 by a gp41-specific human antibody. *Nature* 491 406–412. 10.1038/nature11544 23151583 PMC4854285

[B27] Julg B. Liu P. T. Wagh K. Fischer W. M. Abbink P. Mercado N. B. (2017). Protection against a mixed SHIV challenge by a broadly neutralizing antibody cocktail. *Sci. Transl. Med.* 9:eaao4235. 10.1126/scitranslmed.aao4235 28931655 PMC5747528

[B28] Kaplan H. A. Welply J. K. Lennarz W. J. (1987). Oligosaccharyl transferase: the central enzyme in the pathway of glycoprotein assembly. *Biochim. Biophys. Acta* 906 161–173. 3297152 10.1016/0304-4157(87)90010-4

[B29] Klein F. Gaebler C. Mouquet H. Sather D. N. Lehmann C. Scheid J. F. (2012). Broad neutralization by a combination of antibodies recognizing the CD4 binding site and a new conformational epitope on the HIV-1 envelope protein. *J. Exp. Med.* 209 1469–1479. 10.1084/jem.20120423 22826297 PMC3409500

[B30] Kong L. Ju B. Chen Y. He L. Ren L. Liu J. (2016). Key gp120 glycans pose roadblocks to the rapid development of VRC01-class antibodies in an HIV-1-infected Chinese donor. *Immunity* 44 939–950. 10.1016/j.immuni.2016.03.006 27067056 PMC4862659

[B31] Kwong P. D. Mascola J. R. (2018). HIV-1 vaccines based on antibody identification, B cell ontogeny, and epitope structure. *Immunity* 48 855–871. 10.1016/j.immuni.2018.04.029 29768174

[B32] Li M. Gao F. Mascola J. R. Stamatatos L. Polonis V. R. Koutsoukos M. (2005). Human immunodeficiency virus type 1 env clones from acute and early subtype b infections for standardized assessments of vaccine-elicited neutralizing antibodies. *J. Virol.* 79 10108–10125. 16051804 10.1128/JVI.79.16.10108-10125.2005 PMC1182643

[B33] Li Y. O’Dell S. Walker L. M. Wu X. Mascola J. R. (2011). Mechanism of neutralization by the broadly neutralizing HIV-1 monoclonal antibody VRC01. *J. Virol.* 85 8954–8967. 10.1128/JVI.00754-11 21715490 PMC3165784

[B34] Liao H. X. Lynch R. Zhou T. Gao F. Alam S. M. Boyd S. D. (2013). Co-evolution of a broadly neutralizing HIV-1 antibody and founder virus. *Nature* 496 469–476. 10.1038/nature12053 23552890 PMC3637846

[B35] Lu C. L. Murakowski D. K. Bournazos S. Schoofs T. Sarkar D. Halper-Stromberg A. (2016). Enhanced clearance of HIV-1-infected cells by broadly neutralizing antibodies against HIV-1 in vivo. *Science* 352 1001–1004. 10.1126/science.aaf1279 27199430 PMC5126967

[B36] Lynch R. M. Wong P. Tran L. O’Dell S. Nason M. C. Li Y. (2015). HIV-1 fitness cost associated with escape from the VRC01 class of CD4 binding site neutralizing antibodies. *J. Virol.* 89 4201–4213. 10.1128/JVI.03608-14 25631091 PMC4442379

[B37] Moore P. L. (2018). The neutralizing antibody response to the HIV-1 env protein. *Curr. HIV Res.* 16 21–28. 10.2174/1570162X15666171124122044 29173180 PMC6234226

[B38] Moore P. L. Gray E. S. Morris L. (2009a). Specificity of the autologous neutralizing antibody response. *Curr. Opin. HIV AIDS* 4 358–363. 10.1097/COH.0b013e32832ea7e8 20048698 PMC3004050

[B39] Moore P. L. Ranchobe N. Lambson B. E. Gray E. S. Cave E. Abrahams M. R. (2009b). Limited neutralizing antibody specificities drive neutralization escape in early HIV-1 subtype C infection. *PLoS Pathog.* 5:e1000598. 10.1371/journal.ppat.1000598 19763271 PMC2742164

[B40] Muster T. Steindl F. Purtscher M. Trkola A. Klima A. Himmler G. (1993). A conserved neutralizing epitope on gp41 of human immunodeficiency virus type 1. *J. Virol.* 67 6642–6647. 7692082 10.1128/jvi.67.11.6642-6647.1993 PMC238102

[B41] Pinter A. Honnen W. J. He Y. Gorny M. K. Zolla-Pazner S. Kayman S. C. (2004). The V1/V2 domain of gp120 is a global regulator of the sensitivity of primary human immunodeficiency virus type 1 isolates to neutralization by antibodies commonly induced upon infection. *J. Virol.* 78 5205–5215. 15113902 10.1128/JVI.78.10.5205-5215.2004 PMC400352

[B42] Rademeyer C. Moore P. L. Taylor N. Martin D. P. Choge I. A. Gray E. S. (2007). Genetic characteristics of HIV-1 subtype c envelopes inducing cross-neutralizing antibodies. *Virology* 368 172–181. 17632196 10.1016/j.virol.2007.06.013

[B43] Richman D. D. Wrin T. Little S. J. Petropoulos C. J. (2003). Rapid evolution of the neutralizing antibody response to HIV type 1 infection. *Proc. Natl. Acad. Sci. U.S.A.* 100 4144–4149. 10.1073/pnas.0630530100 12644702 PMC153062

[B44] Robertson D. L. Anderson J. P. Bradac J. A. Carr J. K. Foley B. Funkhouser R. K. (2000). HIV-1 nomenclature proposal. *Science* 288 55–57. 10.1126/science.288.5463.55d 10766634

[B45] Rusert P. Krarup A. Magnus C. Brandenberg O. F. Weber J. Ehlert A. K. (2011). Interaction of the gp120 v1v2 loop with a neighboring gp120 unit shields the HIV envelope trimer against cross-neutralizing antibodies. *J. Exp. Med.* 208 1419–1433. 10.1084/jem.20110196 21646396 PMC3135368

[B46] Sather D. N. Armann J. Ching L. K. Mavrantoni A. Sellhorn G. Caldwell Z. (2009). Factors associated with the development of cross-reactive neutralizing antibodies during human immunodeficiency virus type 1 infection. *J. Virol.* 83 757–769. 10.1128/JVI.02036-08 18987148 PMC2612355

[B47] Simek M. D. Rida W. Priddy F. H. Pung P. Carrow E. Laufer D. S. (2009). Human immunodeficiency virus type 1 elite neutralizers: individuals with broad and potent neutralizing activity identified by using a high-throughput neutralization assay together with an analytical selection algorithm. *J. Virol.* 83 7337–7348. 10.1128/JVI.00110-09 19439467 PMC2704778

[B48] Trkola A. Purtscher M. Muster T. Ballaun C. Buchacher A. Sullivan N. (1996). Human monoclonal antibody 2g12 defines a distinctive neutralization epitope on the gp120 glycoprotein of human immunodeficiency virus type 1. *J. Virol.* 70 1100–1108. 8551569 10.1128/jvi.70.2.1100-1108.1996 PMC189917

[B49] van den Kerkhof T. L. Feenstra K. A. Euler Z. van Gils M. J. Rijsdijk L. W. Boeser-Nunnink B. D. (2013). HIV-1 envelope glycoprotein signatures that correlate with the development of cross-reactive neutralizing activity. *Retrovirology* 10:102. 10.1186/1742-4690-10-102 24059682 PMC3849187

[B50] Van Gils M. J. Bunnik E. M. Boeser-Nunnink B. D. Burger J. A. Terlouw-Klein M. Verwer N. (2011). Longer v1v2 region with increased number of potential n-linked glycosylation sites in the HIV-1 envelope glycoprotein protects against HIV-specific neutralizing antibodies. *J. Virol.* 85 6986–6995. 10.1128/JVI.00268-11 21593147 PMC3126602

[B51] Van Gils M. J. Sanders R. W. (2013). Broadly neutralizing antibodies against HIV-1: templates for a vaccine. *Virology* 435 46–56. 10.1016/j.virol.2012.10.004 23217615

[B52] Walker L. M. Huber M. Doores K. J. Falkowska E. Pejchal R. Julien J. P. (2011). Broad neutralization coverage of HIV by multiple highly potent antibodies. *Nature* 477 466–470. 10.1038/nature10373 21849977 PMC3393110

[B53] Walker L. M. Phogat S. K. Chan-Hui P. Y. Wagner D. Phung P. Goss J. L. (2009). Broad and potent neutralizing antibodies from an African donor reveal a new HIV-1 vaccine target. *Science* 326 285–289. 10.1126/science.1178746 19729618 PMC3335270

[B54] Wang W. Zirkle B. Nie J. Ma J. Gao K. Chen X. S. (2015). N463 glycosylation site on v5 loop of a mutant gp120 regulates the sensitivity of HIV-1 to neutralizing monoclonal antibodies VRC01/03. *J. Acquir. Immune Defic. Syndr.* 69 270–277. 10.1097/QAI.0000000000000595 25751231 PMC4506726

[B55] Wibmer C. K. Bhiman J. N. Gray E. S. Tumba N. Abdool Karim S. S. Williamson C. (2013). Viral escape from HIV-1 neutralizing antibodies drives increased plasma neutralization breadth through sequential recognition of multiple epitopes and immunotypes. *PLoS Pathog.* 9:e1003738. 10.1371/journal.ppat.1003738 24204277 PMC3814426

[B56] Wu X. Kong X. P. (2016). Antigenic landscape of the HIV-1 envelope and new immunological concepts defined by HIV-1 broadly neutralizing antibodies. *Curr. Opin. Immunol.* 42 56–64. 10.1016/j.coi.2016.05.013 27289425 PMC5086270

[B57] Wu X. Wang C. O’Dell S. Li Y. Mascola J. R. (2012). Selection pressure on HIV-1 envelope by broadly neutralizing antibodies to the conserved CD4-binding site. *J. Virol.* 86 5844–5856. 10.1128/JVI.07139-11 22419808 PMC3347292

[B58] Wyatt R. Moore J. Accola M. Desjardin E. Robinson J. Sodroski J. (1995). Involvement of the v1/v2 variable loop structure in the exposure of human immunodeficiency virus type 1 gp120 epitopes induced by receptor binding. *J. Virol.* 69 5723–5733. 10.1016/0166-0934(95)00053-W 7543586 PMC189432

[B59] Zhou T. Georgiev I. Wu X. Yang Z. Y. Dai K. Finzi A. (2010). Structural basis for broad and potent neutralization of HIV-1 by antibody VRC01. *Science* 329 811–817. 10.1126/science.1192819 20616231 PMC2981354

[B60] Zhou T. Lynch R. M. Chen L. Acharya P. Wu X. Doria-Rose N. A. (2015). Structural repertoire of HIV-1-neutralizing antibodies targeting the CD4 supersite in 14 donors. *Cell* 161 1280–1292. 10.1016/j.cell.2015.05.007 26004070 PMC4683157

[B61] Zhu J. Ofek G. Yang Y. Zhang B. Kwong P. D. (2013). Mining the antibodyome for HIV-1-neutralizing antibodies with next-generation sequencing and phylogenetic pairing of heavy/light chains. *Proc. Natl. Acad. Sci. U.S.A.* 110 6470–6475. 10.1073/pnas.1219320110 23536288 PMC3631616

